# Supplementary material for: Elementary Growth Modes provide a molecular description of cellular self-fabrication
Source: PLoS Comput Biol. 2020 Jan 27;16(1):e1007559. doi: 10.1371/journal.pcbi.1007559 (PMC7004393; doi:10.1371/journal.pcbi.1007559)
Supplement: S1 Text — Supplementary text with 4 main parts: 1. A detailed derivation of the growth rate in terms of reaction rates, at balanced growth. Here we give more detailed exposition of the growth rate in terms of reaction rates, and in particular discuss the assumption that the cellular volume is the combined volume of all metabolites, enzymes and ribosomes. 2. Some basic Linear Programming. The essential notions from Linear Programming necessary to follow the proofs in the main text. 3. A single pathway toy model showing the three inherent non- linearities of self-fabrication. A fully worked out example of the theory. We contrast a small self-fabricating model with its simpler non-self-fabricating counterpart, and explore how the additional nonlinear effects present in the self-fabricating models appear, and how they change fluxes, concentrations, growth rates, and their interdependence. 4. Extensions to EGM theory. We discuss extensions to the theory: adding non-enzymatic reactions, adding a positivity constraint for the ribosomal constraint, and adding stress responses. (PDF) [file pcbi.1007559.s001.pdf]

Supplementary Information for Elementary Growth Modes provide a molecular description of cellular self-fabrication

Daan H. de Groot[1], Josephus Hulshof[2], Bas Teusink[1], Frank J. Bruggeman[1], Robert Planqué[1,2,*]

1 Systems Bioinformatics, Amsterdam Institute for Molecules, Medicines & Systems, Vrije Universiteit Amsterdam, De Boelelaan 1087, 1081 HV, Amsterdam, The Netherlands
2 Department of Mathematics, Vrije Universiteit Amsterdam, De Boelelaan 1081a, 1081 HV, Amsterdam, The Netherlands

1 A detailed derivation of the growth rate in terms of reaction rates, at balanced growth

We start with

$$(A1) \quad \dot{n}_k := V \sum_j N_{kj} v_j(\boldsymbol{c}) \quad \text{for all } k. \tag{1}$$

Here N denotes the stoichiometry matrix, and $v_j(\boldsymbol{c})$ is the j-th reaction rate, as a function of concentrations $\boldsymbol{c}$. Since $n_k = Vc_k$, we have

$$\dot{V} c_k + V \dot{c}_k = V \sum_j N_{kj} v_j(\boldsymbol{c}) \quad \text{for all } k. \tag{2}$$

In balanced growth, all concentrations are constant over time (1), $\dot{\boldsymbol{c}} = 0$, which means that

$$\frac{\dot{V}}{V} c_k = \sum_j N_{kj} v_j(\boldsymbol{c}) \quad \text{for all } k. \tag{3}$$

The time derivative of $V = V(\boldsymbol{n})$ is given by

$$\dot{V} = \sum_l \frac{\partial V}{\partial n_l} \dot{n}_l. \tag{4}$$

Hence, using (4) and (1) in (3), we have in balanced growth that

$$\left(\sum_l \frac{\partial V}{\partial n_l} V \sum_j N_{lj} v_j(\boldsymbol{c}) \right) c_k = V \sum_j N_{kj} v_j(\boldsymbol{c}) \quad \text{for all } k. \tag{5}$$

The steady-state assumption ensures that the concentrations $\boldsymbol{c}$, and therefore also the reaction rates $v_j(\boldsymbol{c})$ are constant in time. Therefore, the complete right-hand side of (5) is constant in time, and thus the left-hand side must be as well. This strongly suggests (but does not directly imply) that $\frac{\partial V}{\partial n_l}$ is independent of time as well.[1] Since $\boldsymbol{n}$ does change in time, this implies that $\frac{\partial V}{\partial n_l}$ does not depend explicitly on $\boldsymbol{n}$, i.e.:

$$\frac{\partial V}{\partial n_l} = \rho_l \quad \text{for all } l,$$

for constants ρ_l.[2] The biophysically reasonable assumption that total volume is the sum of all the volumes taken up by individual molecules,

$$(A2) \quad V(\boldsymbol{n}) = \sum_l \rho_l n_l \tag{6}$$

[1]The only alternative would imply that the weighted sum, $\sum_l N_{lj} \frac{\partial V}{\partial n_l}$, is constant in time for all chemical reactions j while the $\frac{\partial V}{\partial n_l}$ are not. This is unlikely.

[2]If we assume that the osmotic pressure is kept constant in a cell, the import of a particle must lead to the import of water. The molar volume parameters, ρ_l, will therefore not only comprise the volume of particle l, but also the volume of the water that is needed to keep the osmotic pressure constant. These parameters thus depend on the osmotic pressure.

is therefore suitable to study balanced growth states. With this definition, we also have

$$\boldsymbol{\rho} \cdot \boldsymbol{c} = \sum_l \rho_l c_l = \frac{1}{V} \sum_l \rho_l n_l = 1 \tag{7}$$

at balanced growth.

The (instantaneous) growth rate is now defined as

$$\mu(t) := \frac{\dot{V}(t)}{V(t)}. \tag{8}$$

Since $\boldsymbol{\rho} \cdot \boldsymbol{c} = 1$ at balanced growth,

$$\begin{aligned}
0 &= \frac{d}{dt}(\boldsymbol{\rho} \cdot \boldsymbol{c}) \\
&= \sum_k \rho_k \dot{c}_k \\
&= \sum_k \rho_k \left(\sum_j N_{kj} v_j(\boldsymbol{c}) - \frac{\dot{V}}{V} c_k \right) \quad \text{using (8)} \\
&= \boldsymbol{\rho} \cdot N\boldsymbol{v}(\boldsymbol{c}) - \boldsymbol{\rho} \cdot (\mu \boldsymbol{c}) \\
&= \boldsymbol{\rho} \cdot N\boldsymbol{v}(\boldsymbol{c}) - \mu(\boldsymbol{\rho} \cdot \boldsymbol{c}) \\
&= \boldsymbol{\rho} \cdot N\boldsymbol{v}(\boldsymbol{c}) - \mu \text{ using (7).}
\end{aligned}$$

We conclude that the growth rate of a cell at balanced growth can be expressed in terms of the catalytic activities of its constituents reactions as,

$$\mu = \boldsymbol{\rho} \cdot N\boldsymbol{v}(\boldsymbol{c}) = \sum_l \rho_l \sum_j N_{lj} v_j(\boldsymbol{c}). \tag{9}$$

2 Some basic Linear Programming

We give a short review of some basic Linear Programming (see e.g. (2; 3)) necessary for the definition of Elementary Growth States and Elementary Growth Modes.

Let A be an $m \times n$ matrix, $m < n$. The linear programs in this paper are all in *equational* or *standard form*

$$\begin{aligned}
&\text{maximize } \boldsymbol{c} \cdot \boldsymbol{x} \\
&\text{subject to } A\boldsymbol{x} = \boldsymbol{b}, \\
&\qquad \boldsymbol{x} \geq \boldsymbol{0}.
\end{aligned}$$

We may assume that A has full rank m (if A does not have full rank the matrix may be reduced to a matrix with fewer rows which does have full rank without changing the solution space of vectors satisfying $A\boldsymbol{x} = \boldsymbol{b}$). A vector $\boldsymbol{x}$ is called a *feasible solution* if it satisfies all the constraints, so $A\boldsymbol{x} = \boldsymbol{b}$ and $\boldsymbol{x} \geq \boldsymbol{0}$.

Let $D \subseteq \{1, \ldots, n\}$ be an index set, and let A_D denote the matrix consisting of columns from A whose indices are in D. We will use similar notation for the restriction of $\boldsymbol{x}$ to the index set D, $\boldsymbol{x}_D$. A vector $\boldsymbol{x}$ is called a *basic feasible solution* if $\boldsymbol{x}$ is a feasible solution for which there exists a set $D \subseteq \{1, \ldots, n\}$ with m distinct elements such that A_D is square and non-singular, $\boldsymbol{x}$ satisfies $A_D \boldsymbol{x}_D = \boldsymbol{b}$, and $x_j = 0$ for all $j \notin D$. The vector $\boldsymbol{x}_D$ is then uniquely defined, since $\boldsymbol{x}_D = A_D^{-1}\boldsymbol{b}$. For any index set D with A_D invertible, we can compute $\boldsymbol{x}_D$. If this solution satisfies $\boldsymbol{x}_D \geq \boldsymbol{0}$, then it induces a basic feasible solution $\boldsymbol{x}$ that satisfies $x_j = 0$ for all indices of $j \notin D$. D is then called a feasible basis for $\boldsymbol{x}$. (This parlance is different from the usual definition of a basis in Linear Algebra, in which a basis is formed by a set of vectors.)

We also recall a few standard concepts from convex geometry. A set $X \subset \mathbb{R}^n$ is called *convex* if for each $\boldsymbol{x}, \boldsymbol{y} \in X$ and $\lambda \in (0, 1)$, we have $\lambda \boldsymbol{x} + (1 - \lambda)\boldsymbol{y} \in X$. A set in $\mathbb{R}^n$ is a *convex polyhedron* if it is an intersection of finitely many closed half-spaces in $\mathbb{R}^n$. A *cone* is a set $X \subset \mathbb{R}^n$ with the property that

$\boldsymbol{x} \in X$ implies $\lambda \boldsymbol{x} \in X$ for all $\lambda \in \mathbb{R}$ and the cone is *pointed* if $\lambda \boldsymbol{x} \in X$ if and only if $\lambda \geq 0$. A *convex polytope* is a bounded convex polyhedron.

A vertex of a convex polyhedron $\mathcal{P} \subset \mathbb{R}^n$ is a point $\boldsymbol{x} \in \mathcal{P}$ such that there exists a vector $\boldsymbol{c}$ with the property that $\boldsymbol{c} \cdot \boldsymbol{x} > \boldsymbol{c} \cdot \boldsymbol{y}$ for all $\boldsymbol{y} \in \mathcal{P} \backslash \{\boldsymbol{x}\}$. The space of feasible solutions of a Linear Program form a convex polyhedron. A standard result from Linear Programming states that the vertices of this polyhedron coincide exactly with the basic feasible solutions of the LP. Lastly, for a cone defined by

$$A\boldsymbol{\alpha} = \mathbf{0}, \quad \boldsymbol{\alpha} \geq \mathbf{0}$$

we define its support by

$$\operatorname{supp} \boldsymbol{\alpha} = \{j \mid \alpha_j \neq 0\}. \tag{10}$$

A standard result from LP is that the spanning rays of such a cone are those vectors in the cone with minimal support.

3 A single pathway toy model showing the three inherent nonlinearities of self-fabrication

We here consider the simplest possible model of a self-fabricator, and compare it with a more conventional model. This will allow us to highlight the inherent nonlinearities of self-fabrication that can be captured with EGM theory. We will then extend this model with an additional step so that we create a core model of overflow metabolism, based on a non-self-fabricating model that we analyzed before (4). These models are not made with the intention to make the most realistic model possible. For this, various more extensive models have already been made by others. See (5) for an overview of all resource-allocation models of overflow metabolism. Instead, our models are meant to illustrate the differences in the mathematical structure of models of self-fabrication compared to conventional models.

The Matlab code used to generate the plots in this section can be found separately as Supporting Information.

3.1 Setting up the stoichiometry and the balanced growth assumption

The first model contains two metabolic reactions: 1) a transporter step that imports the external nutrient and converts it to a metabolite X, and 2) a reaction that generates ATP using this metabolite. In addition, the model contains a ribosome that synthesizes itself and the enzymes needed for the metabolic steps using variable amounts of X and ATP as precursors. This is captured in the following stoichiometry:

$$N = \begin{bmatrix} P & -M \\ \mathbf{0} & I \end{bmatrix}, \quad \text{where } P = \begin{bmatrix} 1 & -1 \\ 0 & 26 \end{bmatrix}, \quad \text{and} \quad M = \begin{bmatrix} 100 & 200 & 210 \\ 800 & 700 & 900 \end{bmatrix}. \tag{11}$$

In non-self-fabricating models the enzyme synthesis is not explicitly modelled. Instead, the demand for precursors is often modelled by a virtual biomass reaction. This reaction needs to be imposed on the model and is often based on experimental data. To compare our self-fabricator model with a non-self-fabricating model, we will assume that the biomass reaction is equal to the mean of the enzyme synthesis reactions, i.e.,

$$N = \begin{bmatrix} P & -M \end{bmatrix}, \text{ where } P = \begin{bmatrix} 1 & -1 \\ 0 & 26 \end{bmatrix}, \quad \text{and} \quad M = \begin{bmatrix} 170 \\ 800 \end{bmatrix}. \tag{12}$$

Note that, since we do not keep track of the production of enzymes and ribosomes anymore, the bottom half of the stoichiometric matrix drops out.

We then add the balanced growth assumption to the model, demanding that all concentrations of all cellular compounds are constant. The net synthesis and consumption of the compounds is captured by the stoichiometry, but on top of that, the compounds also dilute by growth. We thus have

$$P\boldsymbol{v} - M\boldsymbol{w} - \mu\boldsymbol{x} = \mathbf{0}, \qquad \text{and} \qquad I\boldsymbol{w} - \mu\boldsymbol{e} = \mathbf{0}, \tag{13}$$

where $\boldsymbol{v}$ and $\boldsymbol{w}$ are the flux vectors of metabolic reactions (catalyzed by enzymes) and enzyme synthesis reactions (catalyzed by the ribosome), $\boldsymbol{x}$ is the vector of metabolite concentrations, and $\boldsymbol{e}$ is the vector of enzyme concentrations, including the ribosome concentration this time as its last entry.

Linearized models do not include a dilution by growth term. This is probably because metabolite and enzyme concentrations are not explicitly calculated in these models, and the dilution rate is therefore unknown. Instead, a steady-state assumption is used

$$P\boldsymbol{v} - M\boldsymbol{w} = \boldsymbol{0}, \tag{14}$$

where $\boldsymbol{w}$ is now the biomass reaction rate.

3.2 Introducing enzyme/ribosome kinetics

In the model we choose the following rate equations

$$\begin{aligned}
v_{\text{trans}} &= e_{\text{trans}} \frac{glc_{\text{ext}} k_{\text{cat,trans}}}{glc_{\text{ext}} + k_{\text{M,trans}} + \frac{x}{k_{\text{i,X}}}}, \\
v_{\text{resp}} &= e_{\text{resp}} \frac{x k_{\text{cat,resp}}}{x + k_{\text{M,resp}} + \frac{atp}{k_{\text{i,ATP}}}}, \\
w_{\text{synth,trans}} &= r\alpha_{\text{trans}} g_{\text{trans}}(atp, x), \\
w_{\text{synth,resp}} &= r\alpha_{\text{resp}} g_{\text{resp}}(atp, x), \\
w_{\text{synth,rib}} &= r\alpha_{\text{rib}} g_{\text{rib}}(atp, x),
\end{aligned}$$

where we have denoted the concentrations of metabolites by using lowercase letters. The α-variables denote the fractions of the ribosome that are translating the corresponding enzyme. Although we could have gone for a more general choice, for the current model we choose the same rate equation for the synthesis of the different enzymes:

$$g_{\text{trans}} = g_{\text{resp}} = g_{\text{rib}} = \frac{x\, atp\, k_{\text{cat,synth}}}{\frac{1}{k_{\text{M,ATP}} k_{\text{M,X}}} + \frac{x}{k_{\text{M,ATP}}} + \frac{atp}{k_{\text{M,X}}} + atp x}, \tag{15}$$

Here we encounter another difference between the general self-fabricator setup and the conventional models. Models with a virtual biomass reaction implicitly assume that the synthesis of all cellular components is equally fast. In contrast, here we could have picked different rate equations for the synthesis of the different enzymes. If the enzyme concentrations would then change because of a different resource allocation, the average rate of enzyme synthesis by the ribosome would change accordingly.

3.3 A definition of the volume and an induced growth rate expression

We argue in the main text, and in SI1, that the cellular volume should be the sum of the volumes of its components. Dividing the resulting expression for the volume by the volume on both sides gives:

$$1 = \rho_X x + \rho_{\text{ATP}}\, atp + \sigma_{\text{trans}} e_{\text{trans}} + \sigma_{\text{resp}} e_{\text{resp}} + \sigma_{\text{rib}} r, \tag{16}$$

where the ρ, σ-variables are volumetric parameters. For example, ρ_X is the volume of a mole of metabolite X. In the model, we choose: $\rho_X = 0.005, \rho_{\text{ATP}} = 0.0002$, and $\sigma_{\text{trans}} = 0.6538, \sigma_{\text{resp}} = 1.1346, \sigma_{\text{rib}} = 1.2231$. This choice will be motivated shortly.

Under the balanced growth assumption (Equation (13)) the assumption of Equation 16 is equivalent to an expression for the growth rate:

$$\mu = \boldsymbol{\rho} \cdot (N\boldsymbol{v} - M\boldsymbol{w}) + \boldsymbol{\sigma} \cdot \boldsymbol{w} = \sum_{k,j} \rho_k \left(N_{kj} v_j - M_{kj} w_j\right) + \sum_j \sigma_j w_j. \tag{17}$$

We then introduce $a_j = \sum_k \rho_k N_{kj}$ and $b_j = \sigma_j - \sum_k M_{kj} w_j$, such that the expression for the growth rate simplifies to:

$$\mu = \sum_j a_j v_j + b_j w_j. \tag{18}$$

This notation shows that all reactions can directly contribute to the growth rate. The coefficients a_j capture the volume added by the reaction itself, and b_j denotes the volume added by the synthesis reaction of the corresponding enzyme. This added volume is equal to the sum of the volumes of the products minus the sum of the volumes of the substrates. Based on the assumption that chemical reactions do not drastically change the volume of the reagents, we have chosen our ρ, σ-parameters such that the coefficients are close to zero:

$$\begin{aligned} a_{\text{trans}} &= 0.005, & b_{\text{trans}} &= -0.0062, \\ a_{\text{resp}} &= 0.0002, & b_{\text{resp}} &= -0.0054, \\ & & b_{\text{rib}} &= -0.0069 \end{aligned}$$

In a typical balanced growth state, the metabolic fluxes ($\boldsymbol{v}$) are much higher than the enzyme synthesis fluxes ($\boldsymbol{w}$), so with Equation (18) we can see that the transport reaction will contribute most to volume growth. This seems reasonable: "the transport reaction imports volume".

In conventional models the growth rate is often assumed to be proportional to the biomass reaction. In the model of self-fabrication that we consider above, however, the growth rate seems to be proportional to the transport reaction. More generally, as long as the assumption holds that the volumes of the products is approximately equal to the volumes of the substrates for each chemical reaction, the growth rate in self-fabrication models will always be determined by the exchange reactions. Below, we will investigate if defining the growth rate as proportional to the biomass reaction is a good approximation, and when it breaks up.

3.4 The complete model

Our model has now become the following set of differential equations:

$$\begin{aligned} \dot{x} &= e_{\text{trans}} f_{\text{trans}}(glc_{\text{ext}}, x) - e_{\text{resp}} f_{\text{resp}}(x, atp) - (100\alpha_{\text{trans}} + 200\alpha_{\text{resp}} + 210\alpha_{\text{rib}}) r g(x, atp) && - \mu x, \\ \dot{atp} &= 26 e_{\text{resp}} f_{\text{resp}}(x, atp) - (800\alpha_{\text{trans}} + 700\alpha_{\text{resp}} + 900\alpha_{\text{rib}}) r g(x, atp) && - \mu\, atp, \\ \dot{e}_{\text{trans}} &= r\alpha_{\text{trans}} g(x, atp) && - \mu e_{\text{trans}}, \\ \dot{e}_{\text{resp}} &= r\alpha_{\text{resp}} g(x, atp) && - \mu e_{\text{resp}}, \\ \dot{r} &= r\alpha_{\text{rib}} g(x, atp) && - \mu r, \\ \mu &= a_{\text{trans}} e_{\text{trans}} f_{\text{trans}}(glc_{\text{ext}}, x) + a_{\text{resp}} e_{\text{resp}} f_{\text{resp}}(x, atp) \\ &\qquad + r g(x, atp)\left(b_{\text{trans}}\alpha_{\text{trans}} + b_{\text{resp}}\alpha_{\text{resp}} + b_{\text{rib}}\alpha_{\text{rib}}\right). \end{aligned}$$

If we pick the ribosome allocation fractions $\boldsymbol{\alpha}$ and assume that the systems is in a steady-state, the system has 6 equations for 6 variables: $(x, atp, e_{\text{trans}}, e_{\text{resp}}, r, \mu)$. In the main text we solve the last four of these equations and insert them in the first two to get our so-called balanced growth equations. This is however not necessary in order to solve the optimization problem and, to increase legibility, we will not do this here. Even for a model this small, the balanced growth equations in full are unwieldy. Instead, we can now use the three α-variables to maximize the growth rate under the above steady-state conditions and the constraint that $\alpha_{\text{trans}} + \alpha_{\text{resp}} + \alpha_{\text{rib}} \leq 1$. We get

$$\begin{aligned} \max_{\boldsymbol{\alpha}} \quad & \mu, \\ \text{subject to} \quad & \dot{x} = \dot{atp} = \dot{e}_{\text{trans}} = \dot{e}_{\text{resp}} = \dot{r} = 0, \\ & \mu = a_{\text{trans}} v_{\text{trans}} + a_{\text{resp}} v_{\text{resp}} + b_{\text{trans}} w_{\text{trans}} + b_{\text{resp}} w_{\text{resp}} + b_{\text{rib}} w_{\text{rib}}, \\ & \alpha_{\text{trans}} + \alpha_{\text{resp}} + \alpha_{\text{rib}} \leq 1, \\ & \alpha_{\text{trans}}, \alpha_{\text{resp}}, \alpha_{\text{rib}} \geq 0. \end{aligned}$$

3.5 Results

We start by doing a full run of two models: a model of self-fabrication and a non self-fabricating version. For a range of glucose concentrations we find the α's that maximize the growth rate in the first model, and the e's that maximize the biomass production rate in the second model.

We see in Figures S1 and S2 that the model of self-fabrication is indeed well approximated by the conventional model. In the following, we will highlight three nonlinearities that are incorporated in the self-fabrication model. Two of these nonlinearities cannot be seen in the conventional model, and we investigate where these differences show.

3.5.1 Enzyme concentrations and fluxes are no longer proportional

A first nonlinearity that is incorporated in both models is due to the nonlinear enzyme kinetics. The dependency of the enzyme activity on the availability of its substrates and products makes sure that fluxes and enzyme concentrations are no longer forced to be proportional. In Figure S3 we plotted the enzyme concentrations and the corresponding fluxes that were calculated by the model. When nonlinear enzyme kinetics are not modelled this would be a straight diagonal line.

3.5.2 The biomass composition changes

In our model of self-fabrication the synthesis of the enzymes and ribosomes is modelled explicitly. The demand for precursors might vary between different enzymes, which is captured in the M-matrix of Equation (11). If the enzyme concentrations change, the precursor demand can change with it: the model thus calculates the demand for cell synthesis. This effect is visible in our model, although it is a small effect, see Figure S4. In conventional models a virtual biomass reaction is added and therefore the demand for cell synthesis is imposed on the model. This will therefore always be constant over the different growth rates. The effect that is shown is small because of the choice of M-matrix: the synthesis of all enzymes and the ribosome requires more ATP than X. Therefore, the decrease in the concentration of the transport-enzyme and the increase of the concentrations of the respiration-enzyme and the ribosome does not change the precursor demand that much. One could however imagine that, especially in larger models, there could be precursors for which the demand per enzyme synthesis reaction varies more. The change in the precursor demand will then have a larger effect on the model behaviour. We changed the M-matrix to emphasize this changing precursor demand a bit more:

$$M = \begin{bmatrix} 100 & 200 & 210 \\ 800 & 700 & 900 \end{bmatrix} \quad \rightarrow \quad M = \begin{bmatrix} 100 & 226 & 244 \\ 800 & 50 & 50 \end{bmatrix}. \tag{19}$$

In Figure S5 we indeed see that the effect is now more pronounced.

3.5.3 The growth rate is not always proportional to the biomass production rate

In Section 3.3 we discussed the expression for the growth rate that is used in our self-fabrication models:

$$\mu = \sum_j a_j v_j + b_j w_j,$$

where a_j and b_j denote the differences in the summed volume of the products with the summed volumes of the substrates. In principle, each reaction thus contributes to volume growth and hence to growth rate. However, there are two assumptions that we can make in which this simplifies. First, if for each reaction the summed volume of products is approximately the same as the summed volume of the substrates, then a_j and b_j will be negligible except for reactions that transport to or from the cell. Second, if we assume that metabolism is in a steady-state, all volume contributions of created internal compounds will cancel because these compounds will also be degraded. In both cases, only the exchange reactions will explicitly contribute to growth. In a situation where both of these assumptions cannot be made, for example in a dynamic model in which polymers take up more or less volume than their constituents because of their folding properties, our definition of the growth rate will clearly deviate from a growth rate that is defined to be proportional to a biomass reaction.

One could now ask if the rate of the biomass reaction rate is a good approximation of the cellular growth rate when one or both of the assumptions is met? In our base model, of which the results were shown in Figure S1, the cellular growth rate is indeed directly proportional to the total synthesis rate. This is because of the following: in our base model the transport step is the only exchange reaction and therefore the only reaction that contributes to volume growth. If the substrate yield (the ratio of the biomass reaction and the transport reaction) is constant, this implies that the growth rate must be proportional to the biomass reaction rate. The substrate yield is indeed quite constant because almost all imported nutrients will be converted to biomass, because the dilution of metabolites is negligible.

The above argumentation immediately indicates that the biomass reaction rate would not approximate the cellular growth rate if the dilution of metabolites is not negligible. We therefore again changed the M-matrix:

$$M = \begin{bmatrix} 100 & 200 & 210 \\ 800 & 700 & 900 \end{bmatrix} \quad \rightarrow \quad M = \begin{bmatrix} 2 & 4 & 4 \\ 16 & 14 & 18 \end{bmatrix}. \tag{20}$$

We also adjust the volumetric parameters ρ_k, σ_j to keep the summed volume of the products of each reaction more or less equal to the summed volume of the substrates:

$$\begin{bmatrix} \rho_X \\ \rho_{\mathrm{ATP}} \\ \sigma_{\mathrm{trans}} \\ \sigma_{\mathrm{resp}} \\ \sigma_{\mathrm{rib}} \end{bmatrix} = \begin{bmatrix} 0.005 \\ 0.0002 \\ 0.6538 \\ 1.1346 \\ 1.2231 \end{bmatrix} \quad \longrightarrow \quad \begin{bmatrix} \rho_X \\ \rho_{\mathrm{ATP}} \\ \sigma_{\mathrm{trans}} \\ \sigma_{\mathrm{resp}} \\ \sigma_{\mathrm{rib}} \end{bmatrix} = \begin{bmatrix} 0.375 \\ 0.015 \\ 0.99 \\ 1.71 \\ 1.77 \end{bmatrix}. \tag{21}$$

The results are shown in Figure S6. The growth rate and the biomass reaction rate are indeed no longer proportional. This is caused by the large dilution rate of the metabolites.

3.5.4 Solutions are not conserved under multiplication

Elementary Flux Modes are defined up to a multiplicative factor. This means that a steady-state flux vector can always be multiplied by a positive number to give another steady-state flux vector. In conventional resource-allocation models it is often assumed that $v_i = e_i k_{\mathrm{cat},i} f_i(\boldsymbol{x})$, so that the reaction rate is proportional to the enzyme concentration when the metabolite concentrations are kept fixed. This means that a steady-state solution can always be scaled by multiplying all enzyme concentrations by the same factor. Indeed, scalar multiplication of the enzyme concentrations leads to the same multiplication of the reaction rates, and this again leads to a steady state by the aforementioned property of EFMs. For example, assume that $(x_0, atp_0, e_{\mathrm{trans},0}, e_{\mathrm{resp},0}, r_0)$ corresponds to a steady-state solution. In a conventional model, the steady-state equations take the form

$$N\boldsymbol{v} = \boldsymbol{0}. \tag{22}$$

If we keep the metabolite concentrations constant, and multiply all enzyme concentrations by $\lambda \in \mathbb{R}$, we get for the i-th element of $N\boldsymbol{v}$,

$$(N\lambda\boldsymbol{v})_i = \sum_j N_{ij}\lambda e_j f_j(\boldsymbol{x}) = \lambda \sum_j N_{ij} e_j f_j(\boldsymbol{x}) = 0. \tag{23}$$

The above is no longer true in a self-fabrication model. We see in Section 3.4 that even if we keep the metabolite concentrations fixed, a solution cannot be easily scaled to another solution. For example, assume that $(x_0, atp_0, e_{\mathrm{trans},0}, e_{\mathrm{resp},0}, r_0, \mu_0)$ corresponds to one balanced growth state. We thus have

$$\mu = \sum_j a_j v_j + b_j w_j = \sum_j a_j e_j f_j(\boldsymbol{x}) + b_j r \alpha_j g_j(\boldsymbol{x}). \tag{24}$$

If we now multiply the enzyme and the ribosome concentrations by λ, we find that

$$\mu' = \sum_j a_j v_j' + b_j w_j' = \sum_j a_j \lambda e_j f_j(\boldsymbol{x}) + b_j \lambda r \alpha_j g_j(\boldsymbol{x}) = \lambda\mu. \tag{25}$$

The differential equation for the ribosome was

$$\dot{r} = r\alpha_{\mathrm{rib}} g(\boldsymbol{x}) - \mu r = 0, \tag{26}$$

but now becomes

$$\dot{r'} = \lambda r \alpha_{\text{rib}} g(\boldsymbol{x}) - \lambda^2 \mu r \neq 0. \tag{27}$$

Similarly, the steady-state equations for the enzymes and the metabolite concentrations are no longer met. Inspection of these equations would suggest multiplying the α-variables by λ too. However, then the time derivatives of the metabolite concentrations will no longer be zero.

The above shows that, in contrast to conventional models, the reaction rates in self-fabrication models are not proportional to the growth rate within an Elementary Mode. While Elementary Flux Modes could thus be defined up to this multiplicative factor, this is no longer possible for Elementary Growth Modes. This is why the concept of the Elementary Growth States was introduced.

3.6 A two pathway toy model showing overflow metabolism

We now add a fermentation reaction to the model as an alternative for the respiration reaction. It converts the metabolite X in 12 ATP-molecules, instead of the 26 molecules produced by respiration. Furthermore, we set the k_{cat} of the fermentation reaction to be higher than the respiration reaction. These adaptations are based on the model of overflow metabolism that we used in (4). Additionally, we introduce an additional constraint on the concentration of the membrane protein $e_{\text{trans}} \leq 0.2$. This gives the results shown in S7.

This model illustrates that the co-utilization of two Elementary Modes can still be caused by growth rate maximization under two constraints. This extends the results found for EFMs in (4) to EGMs.

3.7 Conclusion

By working out this toy model we have illustrated some important differences between models of self-fabrication and conventional models of metabolism. We have pointed out that 1) enzyme concentrations and fluxes are not necessarily proportional, 2) the biomass composition can change, 3) the growth rate is not necessarily proportional to the biomass production rate, 4) flux ratios in Elementary Modes are not constant over different growth rates and metabolite concentrations.

Moreover, we have also explored under what assumptions the above nonlinearities are approximately linear again. This is important because the conventional models were highly successful in fitting experimental data, but less biologically accurate in terms of their mechanistic underpinnings. To reproduce the experimental data with a model of self-fabrication we need some additional assumptions, for example that the biomass composition does not change drastically over different conditions. This thus indicates the importance of assumptions that were first taken for granted.

4 Extensions of EGM theory

4.1 Including non-enzymatic reactions

The metabolic ODEs are now augmented with non-enzymatic reactions with rate vector $\boldsymbol{u}(\boldsymbol{x})$, and stoichiometry matrix S, so that

$$\dot{x}_k = \sum_j S_{kj} u_j(\boldsymbol{x}) + \sum_j P_{kj} e_j f_j(\boldsymbol{x}) - \sum_j M_{kj} r \alpha_j g_j(\boldsymbol{x}) - \mu x_k.$$

Setting $c_j = \sum_k \rho_k S_{kj}$, the definition of μ may be succinctly phrased as

$$\mu = \boldsymbol{c} \cdot \boldsymbol{u} + \boldsymbol{a} \cdot \boldsymbol{v} + \boldsymbol{b} \cdot \boldsymbol{w}.$$

The steady state equations for $\boldsymbol{x}$ now read

$$S\boldsymbol{u} + P\boldsymbol{v} - M\boldsymbol{w} = \boldsymbol{x}(\boldsymbol{c} \cdot \boldsymbol{u} + \boldsymbol{a} \cdot \boldsymbol{v} + \boldsymbol{b} \cdot \boldsymbol{w}).$$

Using the same line of reasoning as before, Eq (21) in the main text becomes

$$r\left(x_k\left[\sum_{j=1}^{n}\left(\frac{a_j f_j(\boldsymbol{x})}{\mu}+b_j\right)\alpha_j(\boldsymbol{x})+b_{n+1}\mu\right]-\sum_{j=1}^{n}\left(\frac{P_{kj} f_j(\boldsymbol{x})}{\mu}+M_{kj}\right)\alpha_j(\boldsymbol{x})+M_{k(n+1)}\mu\right)=\sum_{j=1}^{n}(S_{kj}-x_k c_j)u_j(\boldsymbol{x}). \quad (28)$$

Note that the ribosome concentration now appears in the equations, since it occurs on the left hand side, but not on the right due to the nonenzymatic reactions.

4.2 Including a positivity constraint for the ribosome concentration

The balanced growth equations were derived by first solving the steady state ribosome and enzyme equations, and substituting the result into the steady state equations for the metabolites. Since $\dot{r} = r(\alpha_{n+1} - \mu)$, one does not get immediate information about r in steady state. Since r eventually drops out of the balanced growth equations, because it is a factor in all of them, one needs to derive the steady state ribosome concentration a posteriori. The expression for r is given in Eq (19) in the main text,

$$r = \frac{\mu}{\sum_{j=1}^{n}\left(\frac{a_j f_j(\boldsymbol{x})}{\mu}+\sigma_j-b_j\right)\alpha_j+(\sigma_{n+1}-b_{n+1})\mu}. \quad (29)$$

Since finding any balanced growth solution generally starts with prescribing some metabolite concentration vector $\boldsymbol{x}$, it is not guaranteed that the resulting ribosome concentration is positive. If it is not, the enzyme concentrations in steady state are all negative as well, since $e_j = r\alpha_j/\mu$.

For certain choices of $\boldsymbol{x}$, and growth rate μ, some vectors in the polytope $\mathcal{P}_{\boldsymbol{x},\mu}$ may correspond to negative ribosome and enzyme concentrations. These should of course be discarded. One could incorporate an additional constraint into the polytope, requiring that the denominator of (29) be positive,

$$\sum_{j=1}^{n}\left(\frac{a_j f_j(\boldsymbol{x})}{\mu}+\sigma_j-b_j\right)\alpha_j+(\sigma_{n+1}-b_{n+1})\mu \geq 0.$$

This constraint is again of the same nature as the ones in $B(\boldsymbol{x},\mu)\boldsymbol{\alpha} = \mu\boldsymbol{u}_{m+1}$ in the definition of $\mathcal{P}_{\boldsymbol{x},\mu}$. Choosing a metabolite vector $\boldsymbol{x}$ such that this constraint is violated for all $\boldsymbol{\alpha} \in \mathcal{P}_{\boldsymbol{x},\mu}$ implies that the polytope to which the ribosomal constraint is add would be empty.

Any biologically reasonable balanced growth solution would have a finite, strictly positive ribosome concentration. Therefore, such solutions will not hit this new constraint.

4.3 Including stress responses

Investments in stress responses are often viewed as orthogonal to investments in growth. In EGM theory, this is not necessary. For example, a heat shock response might involve increased expression of chaperone proteins that accelerate the refolding of denaturated proteins. Chaperones themselves do not contribute to the catabolic or anabolic parts of metabolism, and are therefore viewed as not contributing to growth. The chaperones, however, extend the lifetime of proteins (they lower their natural degradation rate, which we have ignored in the whole-cell model but can be put in without any modification of the main results). In this indirect way, chaperones of course increase the growth rate relative to the situation in which they are absent.

An example implementation would be as follows. First we incorporate degradation rates of enzymes,

$$\dot{e}_j = r\alpha_j - \mu e_j - d_j(T,\boldsymbol{h})e_j$$

in which $d_j(T,\boldsymbol{h})$ are the degradation rate of enzymes, as a function of temperature (denaturation) and chaperone concentrations $\boldsymbol{h}$. Next, the chaperones need to be synthesized

$$\dot{h}_k = r\alpha_k - \mu h_k,$$

Variable/ parameter	Description	Unit
$\boldsymbol{n}$	copy number of cellular compounds	mol
$\boldsymbol{c}$	concentration of cellular compounds	molL^{-1}
$\boldsymbol{x}$	concentration of metabolites	molL^{-1}
$\boldsymbol{e}$	concentration of enzymes	molL^{-1}
r	concentration of ribosome	molL^{-1}
α_j	ribosomal fraction allocated to produce enzyme/ribosome	n.a.
N	overall stoichiometric matrix	n.a.
P	metabolic stoichiometric matrix	n.a.
M	metabolite-to-enzyme stoichiometric matrix	n.a.
I	identity matrix	n.a.
μ	growth rate	s^{-1}
ρ_k	volumetric parameter for metabolic compound k	mol^{-1}L
σ_k	volumetric parameter for enzymes and ribosome	mol^{-1}L
f_j	enzymatic rate law	s^{-1}
g_j	enzyme synthesis rate law	s^{-1}
v_j	metabolic rate	mol$L^{-1}s^{-1}$
w_j	enzyme synthesis rate	mol$L^{-1}s^{-1}$
a_j	net contribution to growth rate by metabolic reaction j	mol^{-1}L
b_j	net contribution to growth rate by enzyme/ribosome synthesis reaction j	mol^{-1}L
$\mathcal{C}_{\boldsymbol{x},\mu}$	cone corresponding to metabolite concentration $\boldsymbol{x}$ and growth rate μ	n.a.
$\mathcal{P}_{\boldsymbol{x},\mu}$	polytope corresponding to metabolite concentration $\boldsymbol{x}$ and growth rate μ	n.a.
$\boldsymbol{u}_j$	j-th elementary unit vector	n.a.

Table 1: Overview of all variables and parameters used in this paper.

(here modelled without its own degradation rate, but one could put that in) then one would obtain a new whole cell model, with extended stoichiometry for all the enzymes and now also chaperones. The number of α_j increases: the ribosome now needs to be allocated not only over all enzymes and the ribosome, but also over the chaperones.

If the degradation depends linearly on the chaperone concentration, the resulting balanced growth equations would have the same qualitative properties as before. The polytope $\mathcal{P}_{\boldsymbol{x},\mu,T}$ of all balanced growth solutions at metabolite concentrations $\boldsymbol{x}$ with growth rate μ and temperature T now has new vertices, which are again called EGMs. Depending on T, the maximal growth rate solution may have a positive α_k for the chaperones. In that case, the investment of synthesising chaperones leads to a higher growth rate than if this investment were not made.

Similar implementations may be given for other stress responses. A second example is cleaning up of toxins (see Fig 6 in the main text, and code below), either made as an inevitable byproduct of certain metabolic reactions, or passively diffuse into the cell. Such toxins could for instance lower the k_{cat} of metabolic reactions, leading to a lower growth rate. As long as cleaning up such toxins using specific proteins is implemented using kinetics that are linear in those protein concentrations, the EGM theory applies. If the effect of the toxin is sufficiently detrimental to metabolic reaction rates, the growth rate maximiser will feature investment into proteins that naturalise those toxins.

References

[1] Schaechter M. A brief history of bacterial growth physiology. Frontiers in microbiology. 2015;6:289.

[2] Matoušek J, Gärtner B. Understanding and Using Linear Programming. Springer Verlag, New York; 2007.

[3] Bertsimas D, Tsitsiklis JN. Introduction to Linear Optimization. Athene Scientific, Belmont, Massachusetts; 1997.

[4] de Groot DH, Planqué R, van Boxtel C, Bruggeman FJ, Teusink B. The number of active metabolic pathways is bounded by the number of cellular constraints at maximal metabolic rates. PLoS Comp Biol. 2019;15(3):e1006858.

[5] de Groot DH, Liscke J, Muolo R, Planqué R, Bruggeman FJ, Teusink B. The common message of constraint-based optimization approaches: overflow metabolism is caused by two growth-limiting constraints; 2019.
