# Supplementary material for: Elementary Growth Modes provide a molecular description of cellular self-fabrication
Source: PLoS Comput Biol. 2020 Jan 27;16(1):e1007559. doi: 10.1371/journal.pcbi.1007559 (PMC7004393; doi:10.1371/journal.pcbi.1007559)
Supplement: S1 Table — (PDF) [file pcbi.1007559.s002.pdf]

Variable/ parameter	Description	Unit
$\boldsymbol{n}$	copy number of cellular compounds	mol
$\boldsymbol{c}$	concentration of cellular compounds	molL^{-1}
$\boldsymbol{x}$	concentration of metabolites	molL^{-1}
$\boldsymbol{e}$	concentration of enzymes	molL^{-1}
r	concentration of ribosome	molL^{-1}
α_j	ribosomal fraction allocated to produce enzyme/ribosome	n.a.
N	overall stoichiometric matrix	n.a.
P	metabolic stoichiometric matrix	n.a.
M	metabolite-to-enzyme stoichiometric matrix	n.a.
I	identity matrix	n.a.
μ	growth rate	s^{-1}
ρ_k	volumetric parameter for metabolic compound k	mol^{-1}L
σ_k	volumetric parameter for enzymes and ribosome	mol^{-1}L
f_j	enzymatic rate law	s^{-1}
g_j	enzyme synthesis rate law	s^{-1}
v_j	metabolic rate	mol$L^{-1}s^{-1}$
w_j	enzyme synthesis rate	mol$L^{-1}s^{-1}$
a_j	net contribution to growth rate by metabolic reaction j	mol^{-1}L
b_j	net contribution to growth rate by enzyme/ribosome synthesis reaction j	mol^{-1}L
$\mathcal{C}_{\boldsymbol{x},\mu}$	cone corresponding to metabolite concentration $\boldsymbol{x}$ and growth rate μ	n.a.
$\mathcal{P}_{\boldsymbol{x},\mu}$	polytope corresponding to metabolite concentration $\boldsymbol{x}$ and growth rate μ	n.a.
$\boldsymbol{u}_j$	j-th elementary unit vector	n.a.

Table 1: Overview of all variables and parameters used in this paper.
