# Supplementary figures and images for: Elementary Growth Modes provide a molecular description of cellular self-fabrication
Source: PLoS Comput Biol. 2020 Jan 27;16(1):e1007559. doi: 10.1371/journal.pcbi.1007559 (PMC7004393; doi:10.1371/journal.pcbi.1007559)

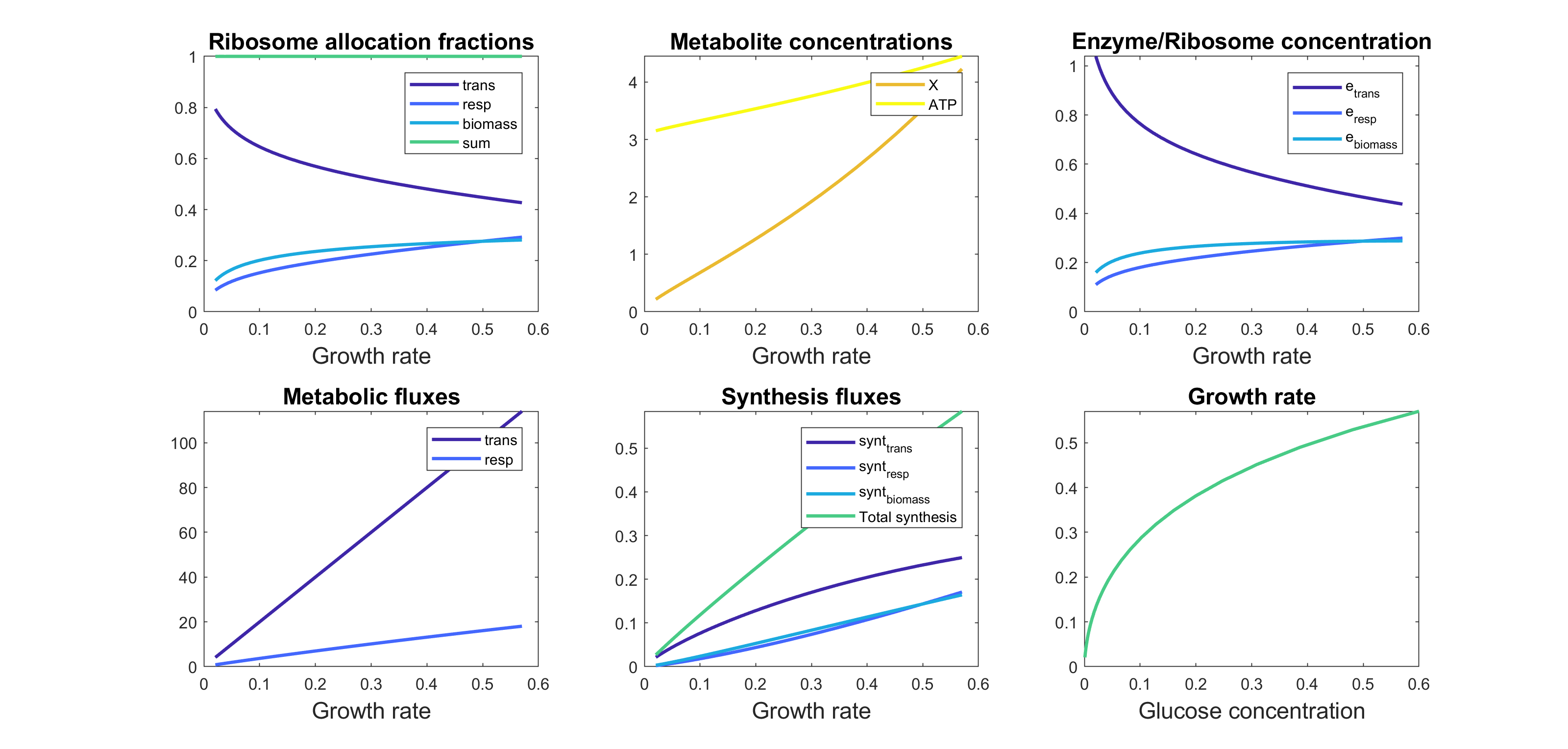

Supplement: S1 Fig — All results reflect optimal allocation for maximal growth rate. (TIF) [file pcbi.1007559.s003.tif]

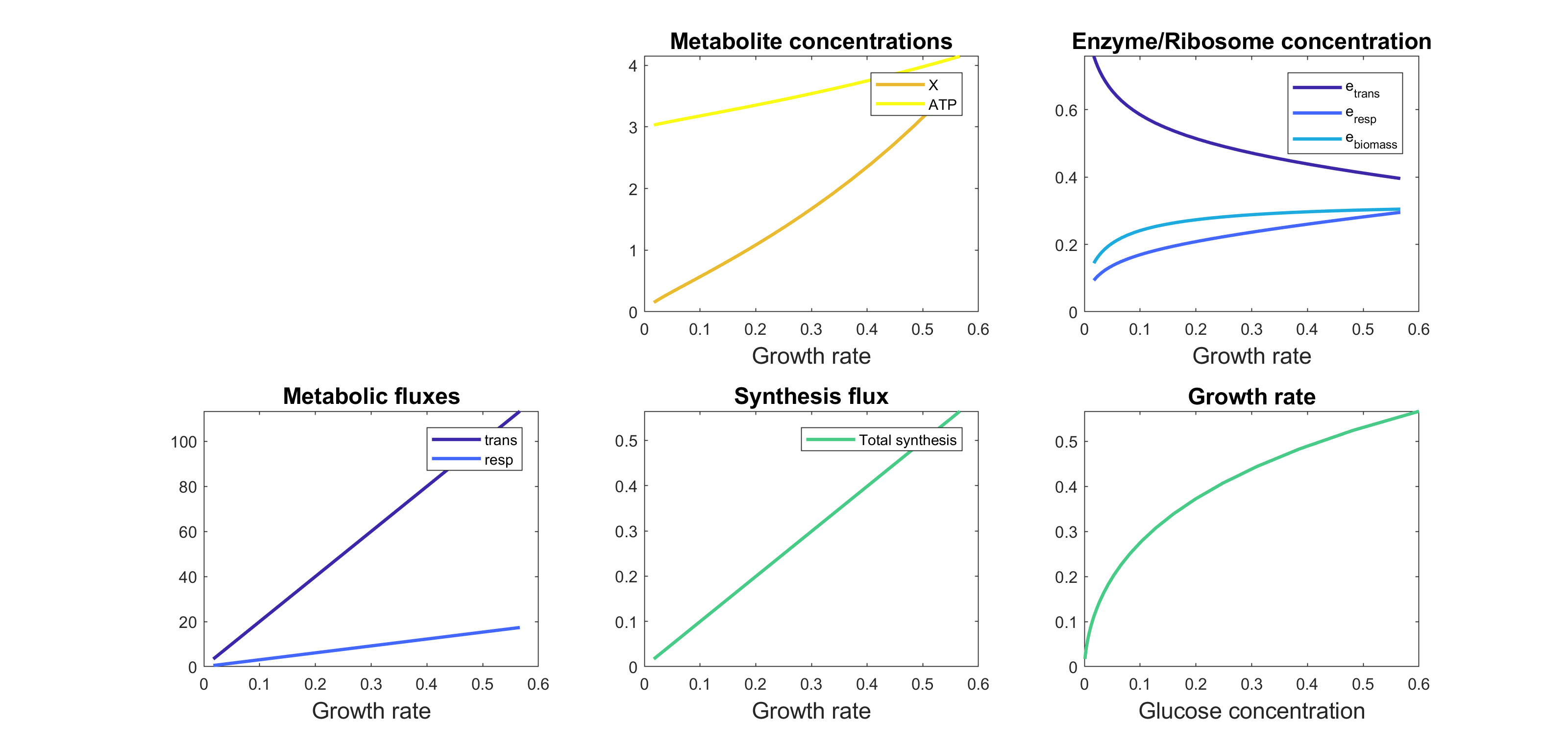

Supplement: S2 Fig — All results reflect optimal allocation for maximal biomass production rate. (TIF) [file pcbi.1007559.s004.tif]

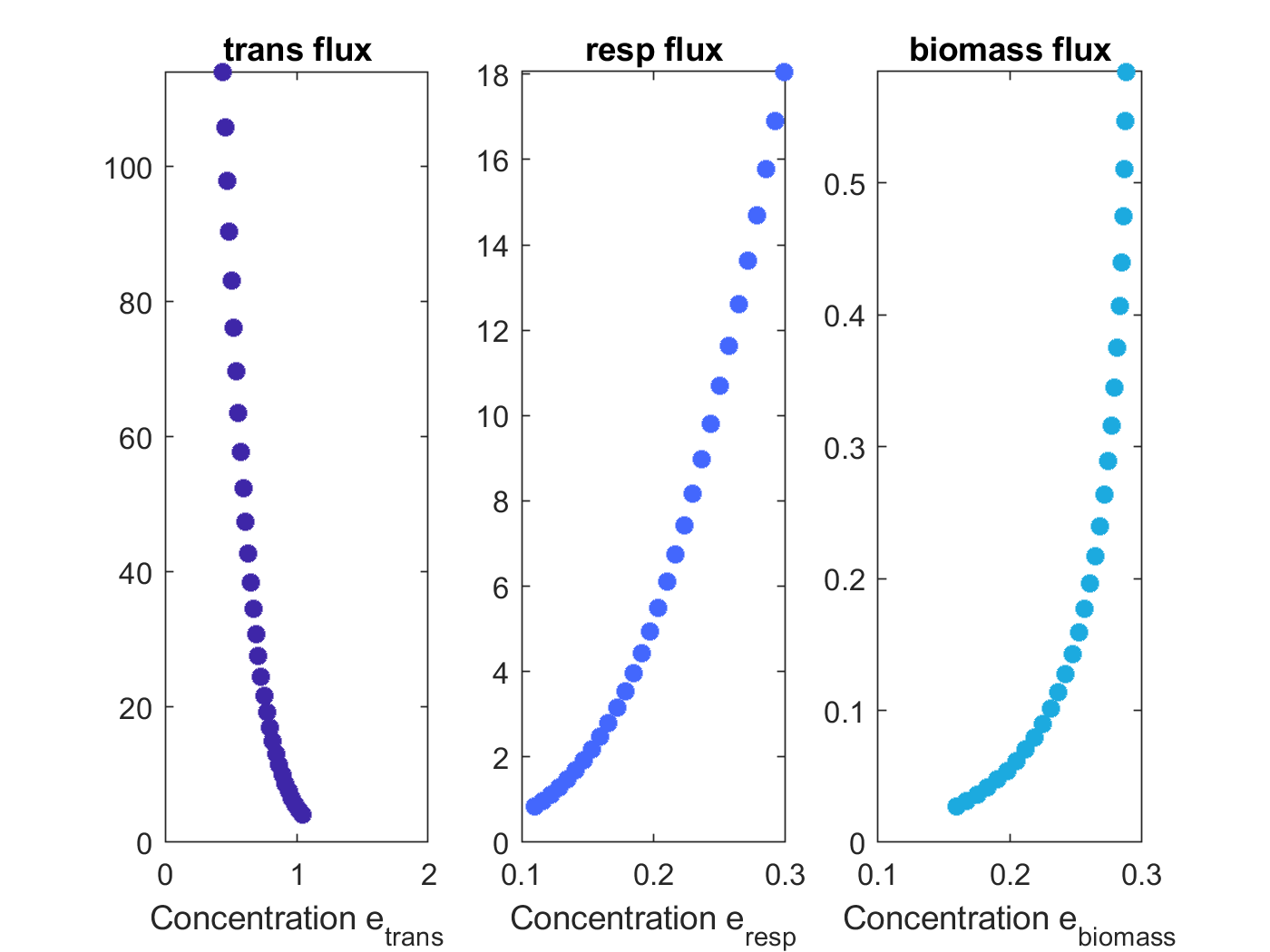

Supplement: S3 Fig — Values shown correspond to optimal growth rate solutions of the model. (TIF) [file pcbi.1007559.s005.tif]

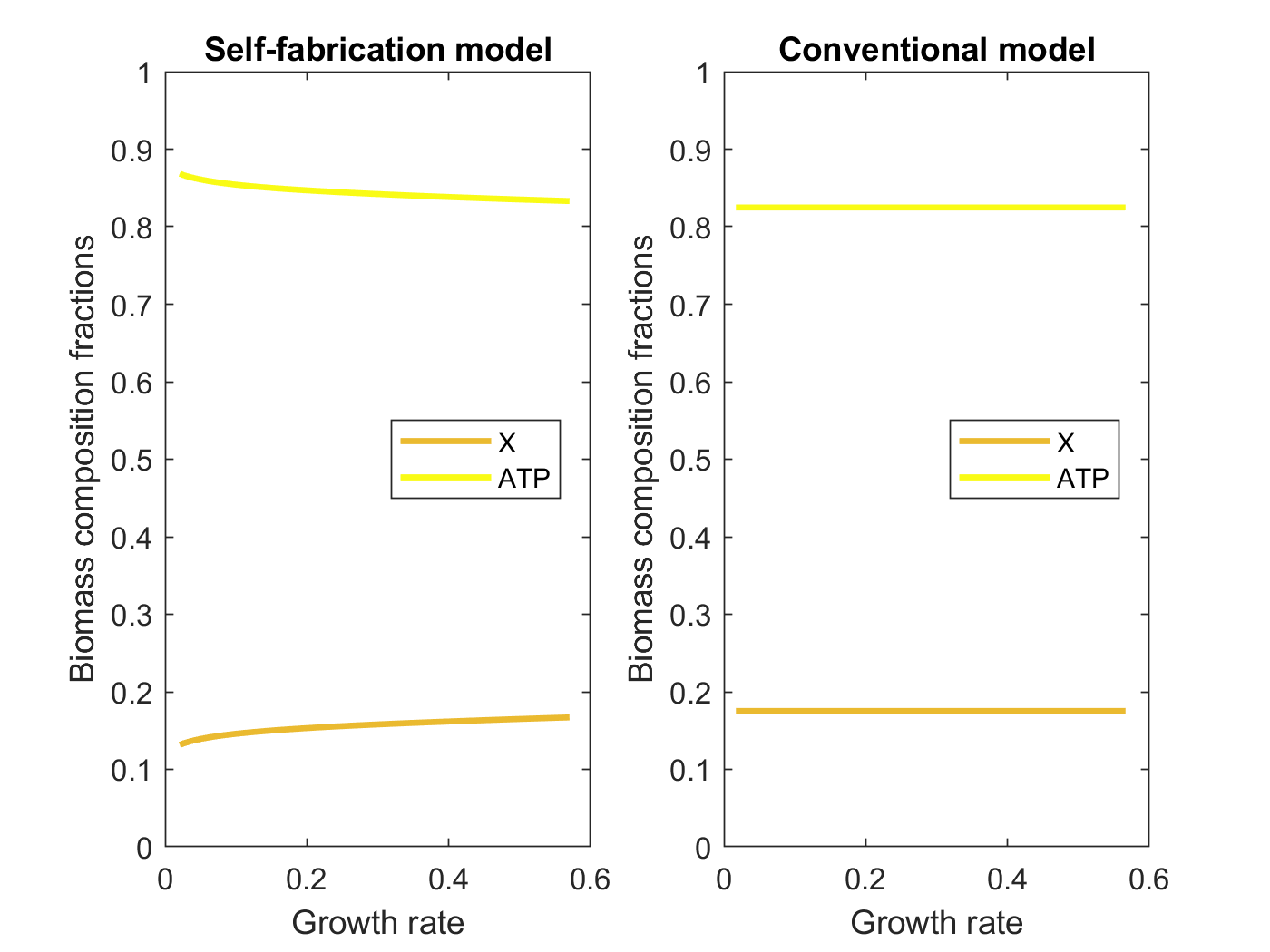

Supplement: S4 Fig — The left figure shows the results for our toy model of self-fabrication, while the right figure shows the results for the corresponding conventional model. (TIF) [file pcbi.1007559.s006.tif]

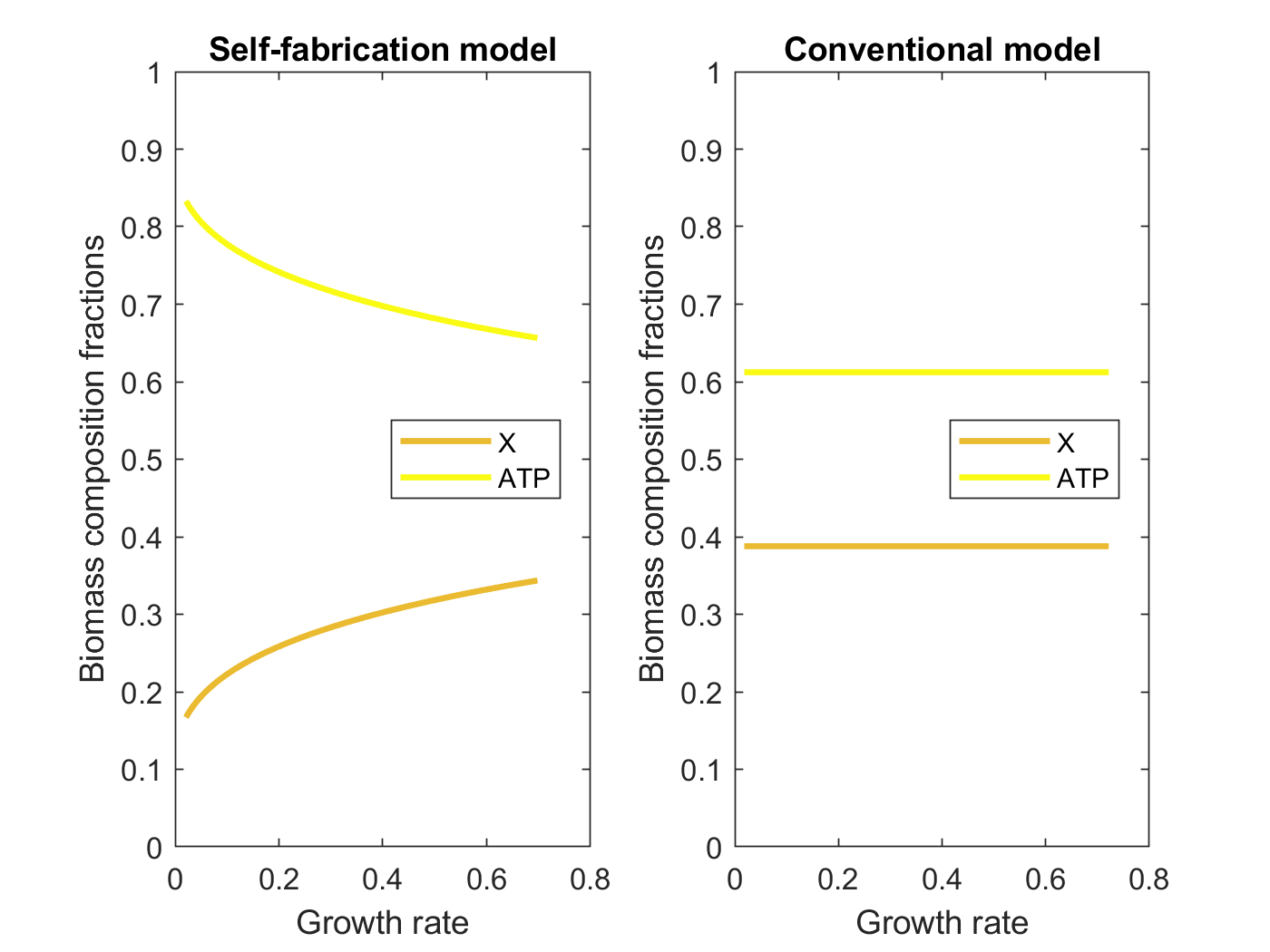

Supplement: S5 Fig — Compared to the model results presented in S4 Fig, there is more variation in the precursor demand for protein and ribosomes. The left figure shows the results for our toy model of self-fabrication, while the right figure shows the results for the corresponding conventional model. (TIF) [file pcbi.1007559.s007.tif]

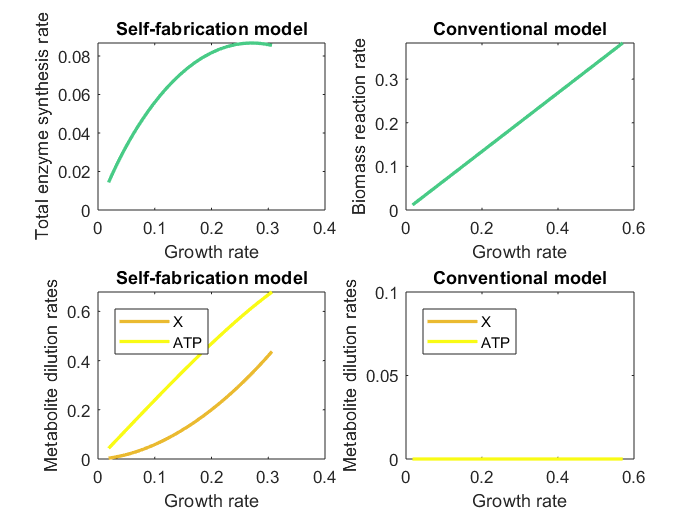

Supplement: S6 Fig — If the dilution rate of metabolite is no longer negligible compared to their metabolic turnover, the growth rate can increase without a proportional increase of the enzyme synthesis. (TIF) [file pcbi.1007559.s008.tif]

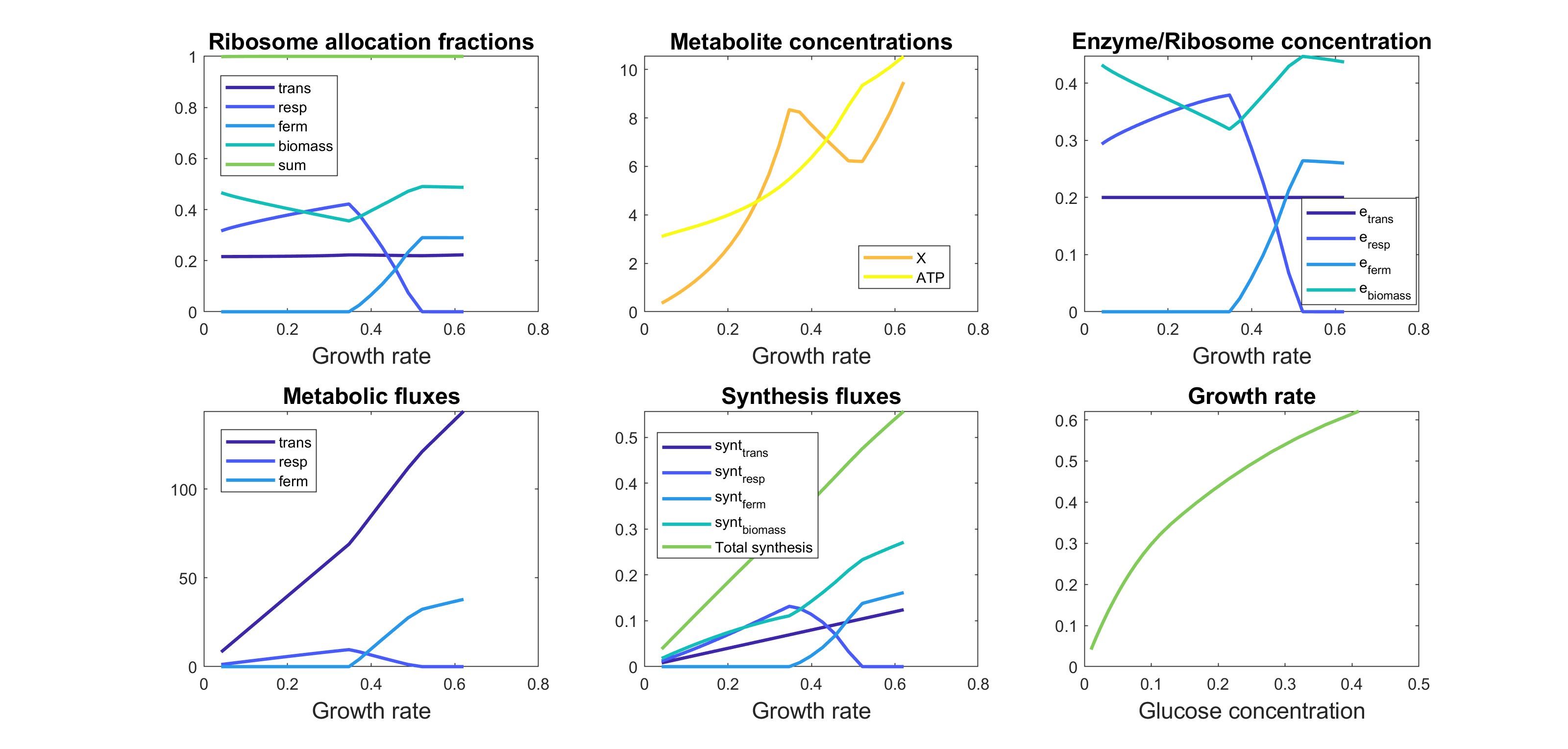

Supplement: S7 Fig — (TIF) [file pcbi.1007559.s009.tif]

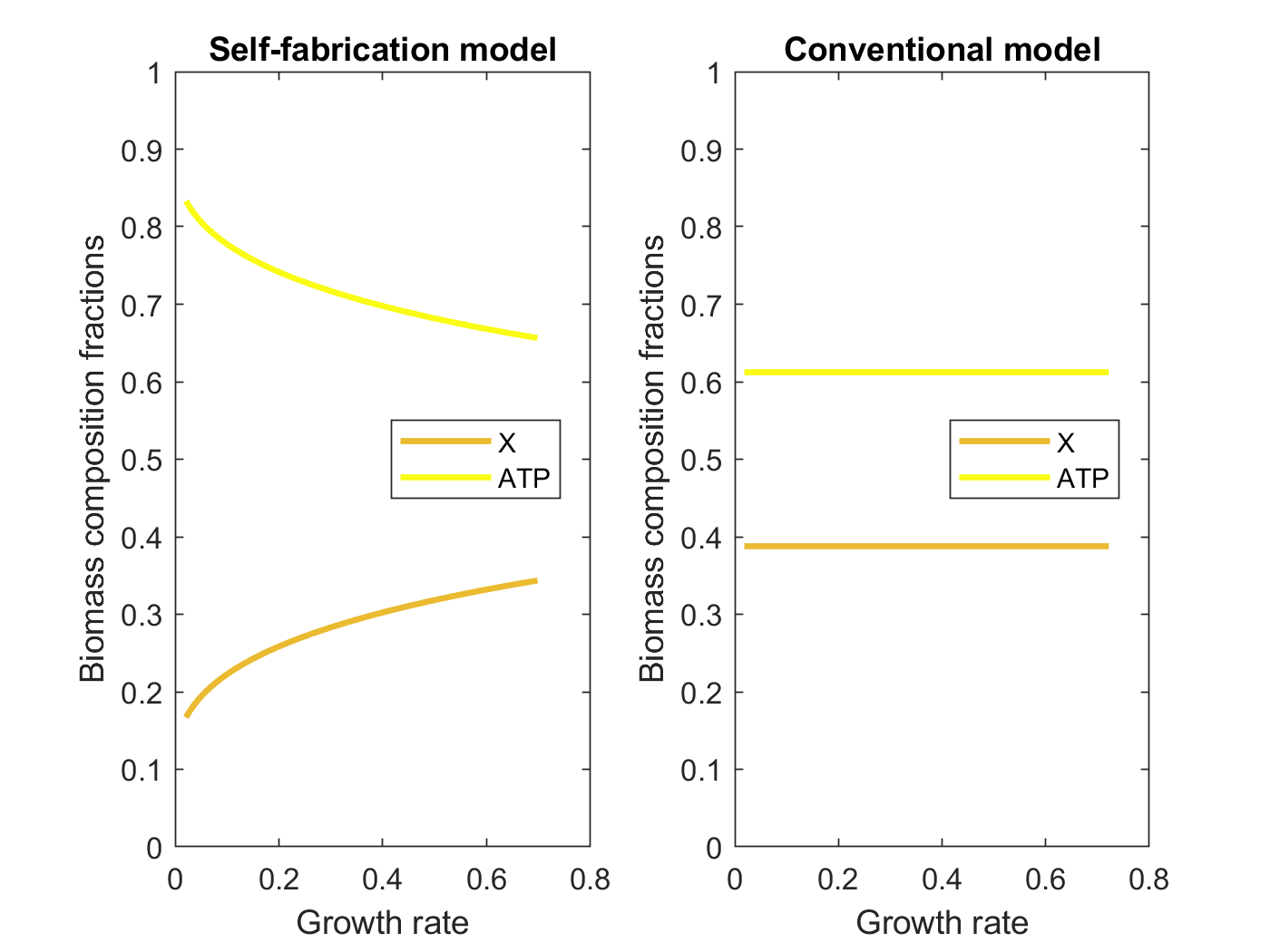

Supplement: S2 Code — (ZIP) [file pcbi.1007559.s011.zip › figures/biomass_composition_emphasized.png]

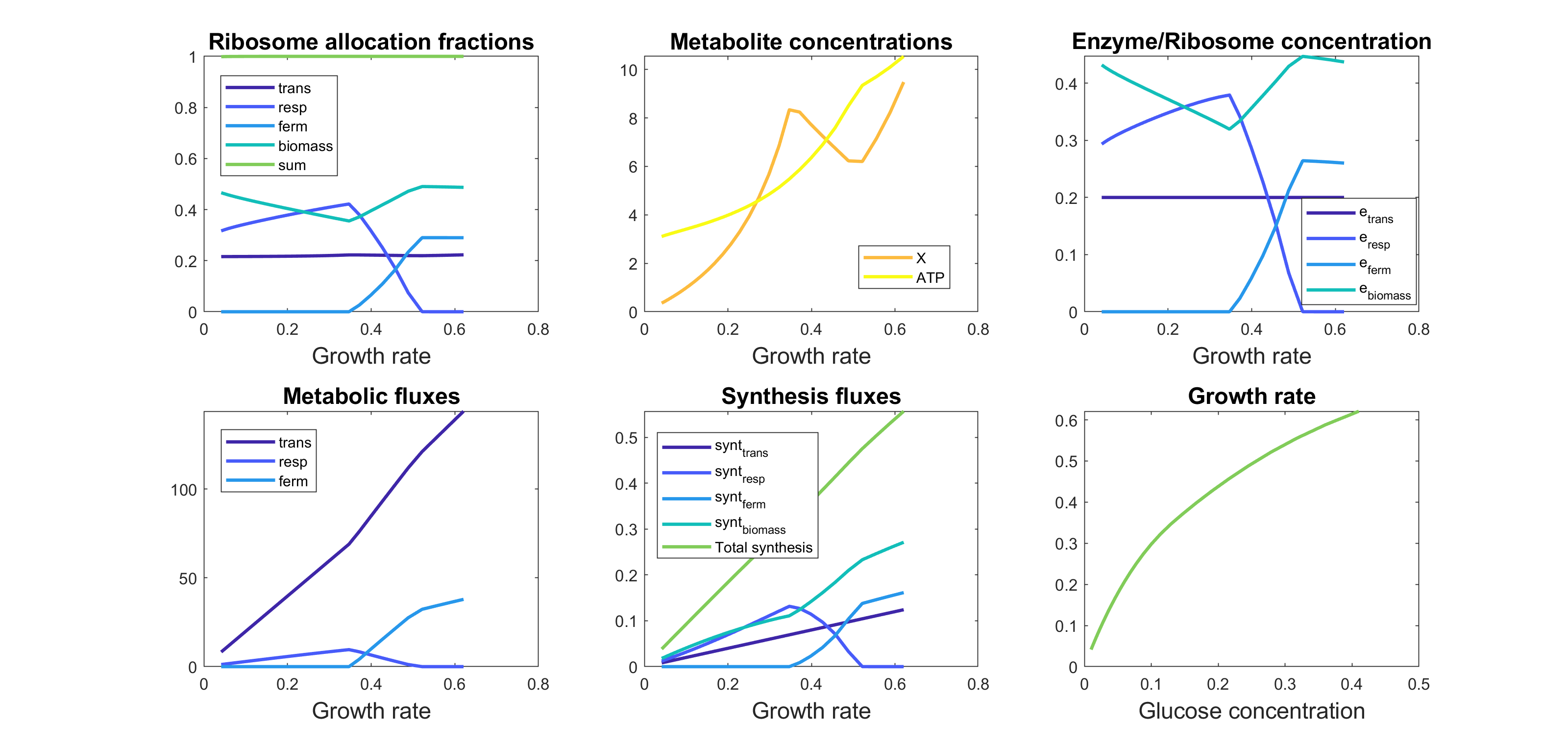

Supplement: S2 Code — (ZIP) [file pcbi.1007559.s011.zip › figures/overflow_self_fab.png]

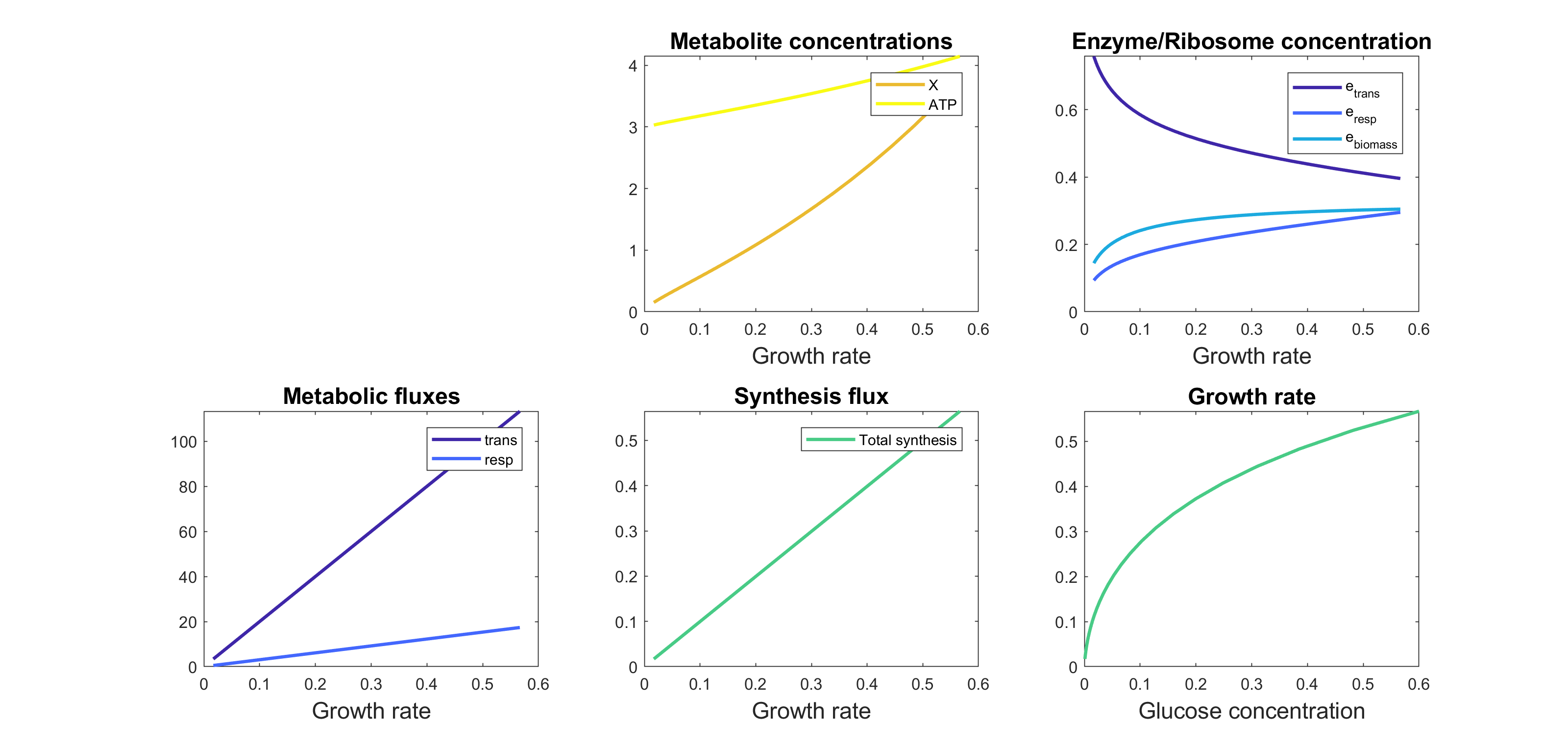

Supplement: S2 Code — (ZIP) [file pcbi.1007559.s011.zip › figures/conc_flux_model_base_n_30_non_sf.png]

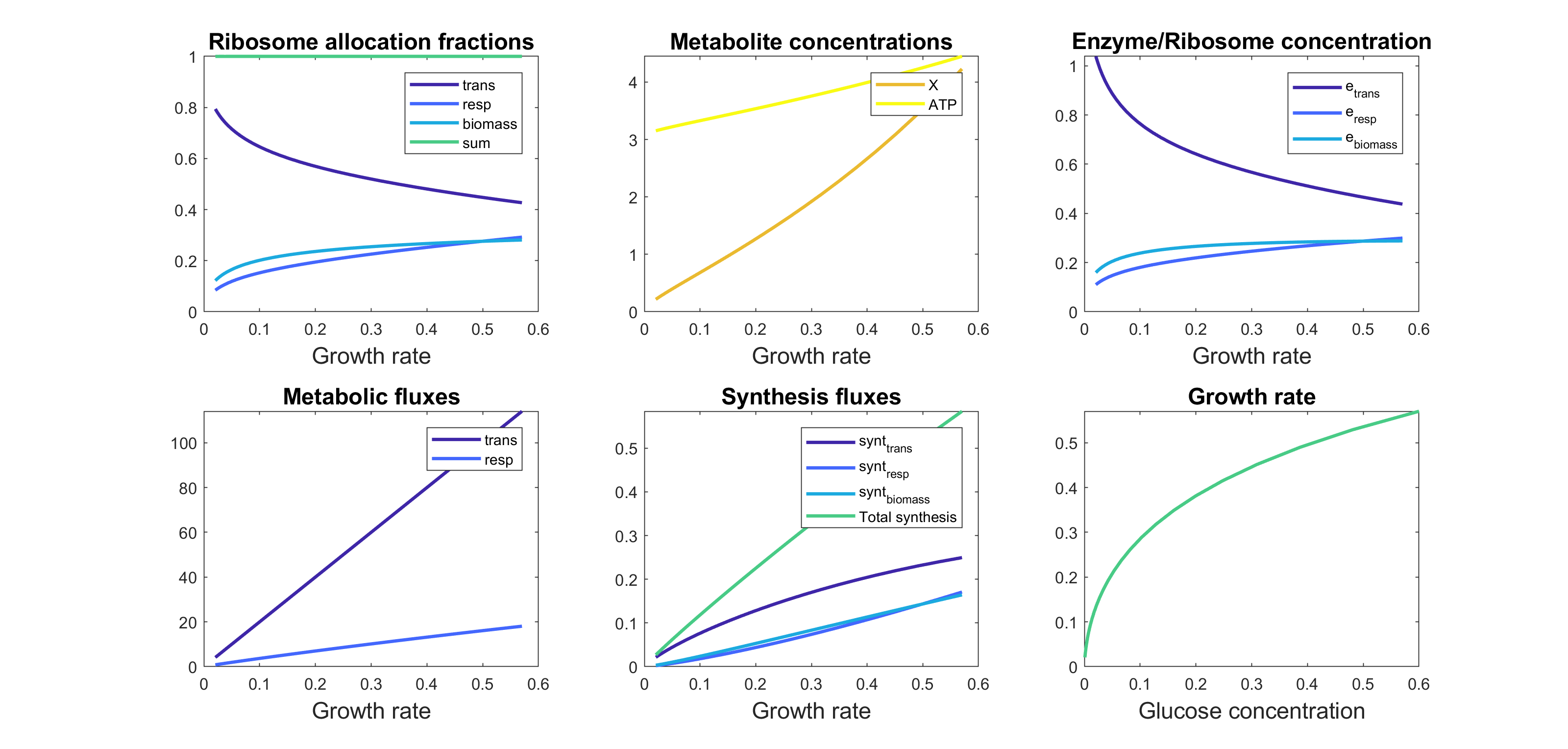

Supplement: S2 Code — (ZIP) [file pcbi.1007559.s011.zip › figures/conc_flux_model_base_n_30.png]

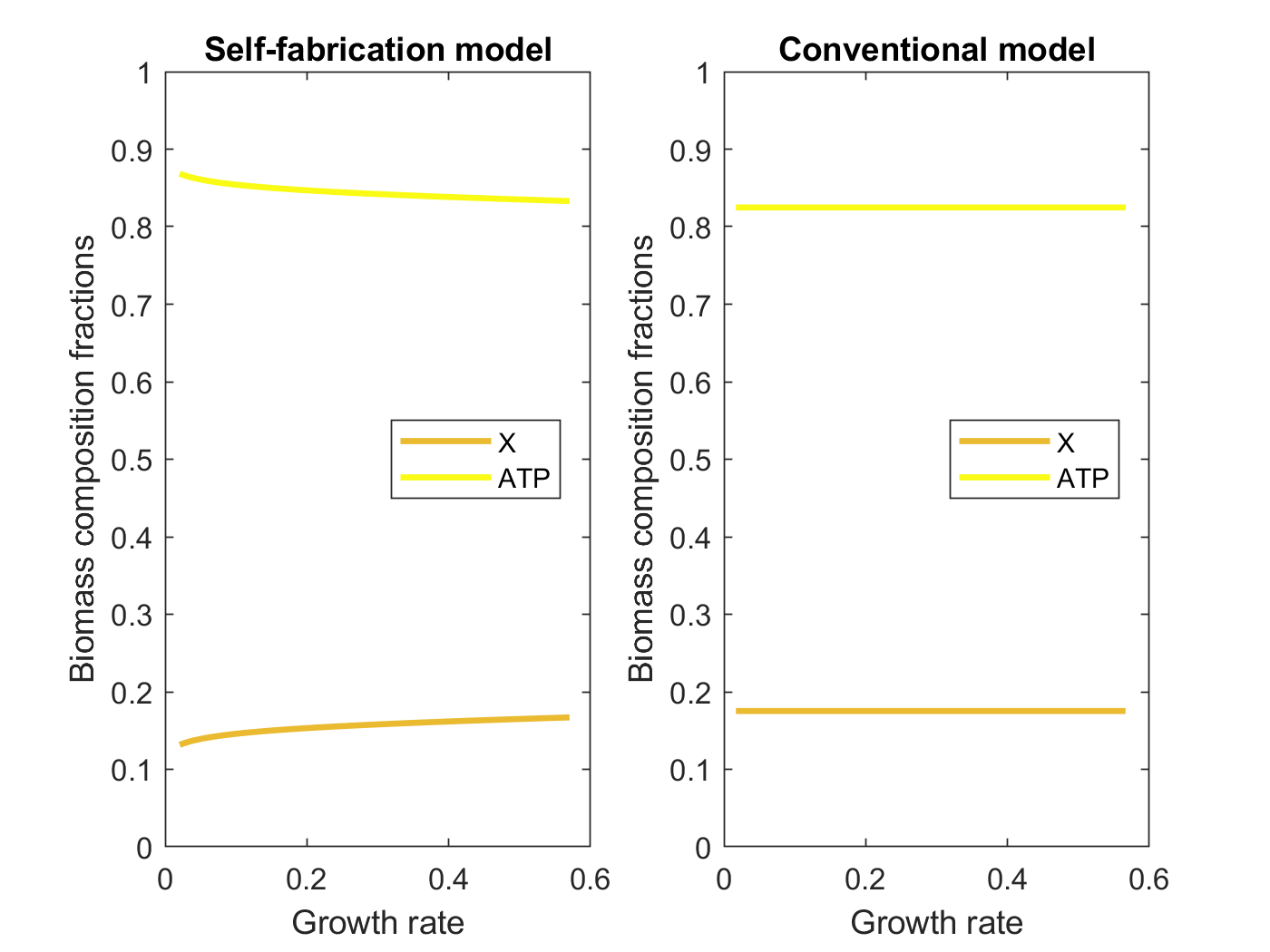

Supplement: S2 Code — (ZIP) [file pcbi.1007559.s011.zip › figures/biomass_composition_non_emphasized.png]

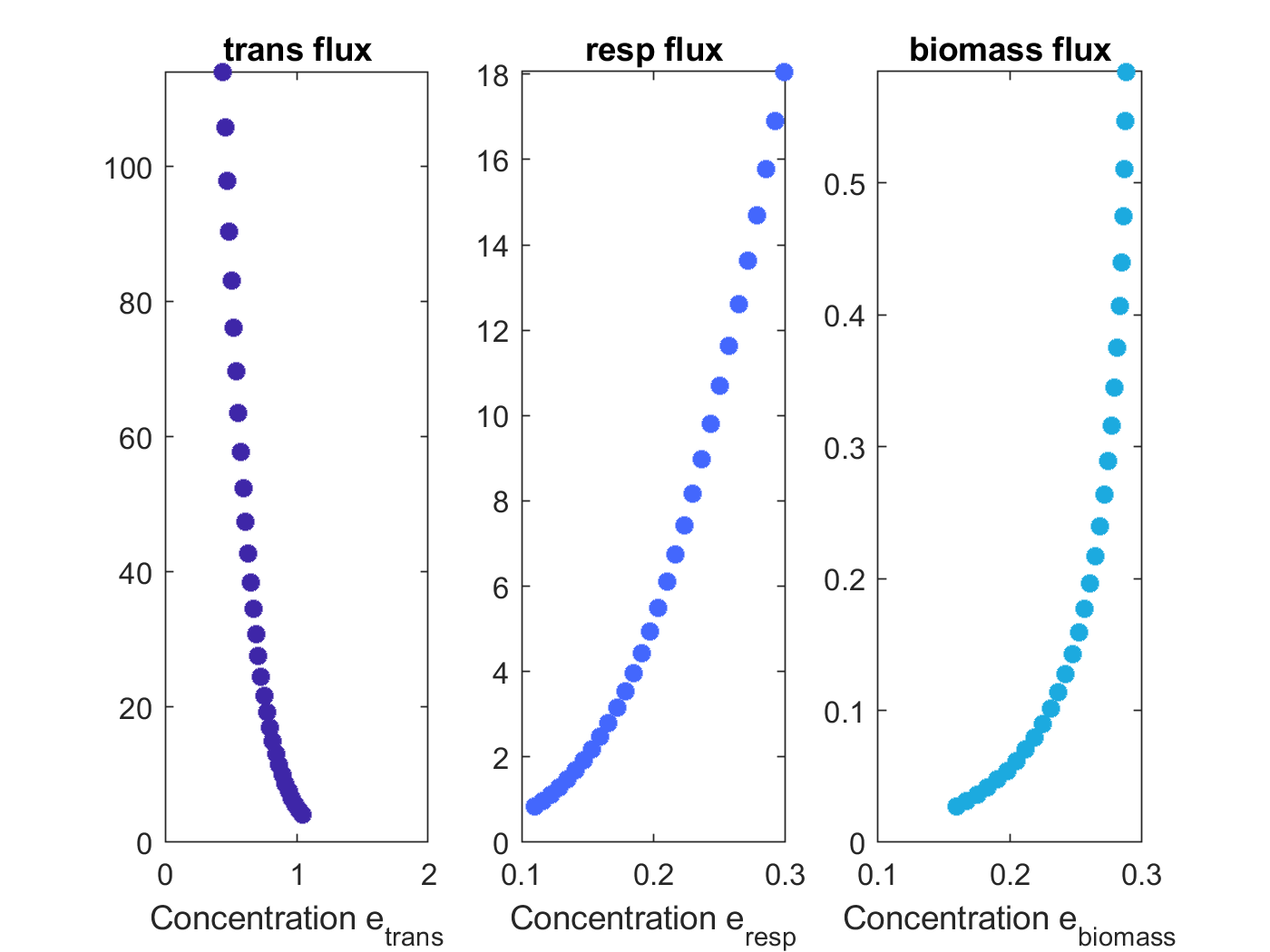

Supplement: S2 Code — (ZIP) [file pcbi.1007559.s011.zip › figures/enzyme_flux_not_proportional.png]

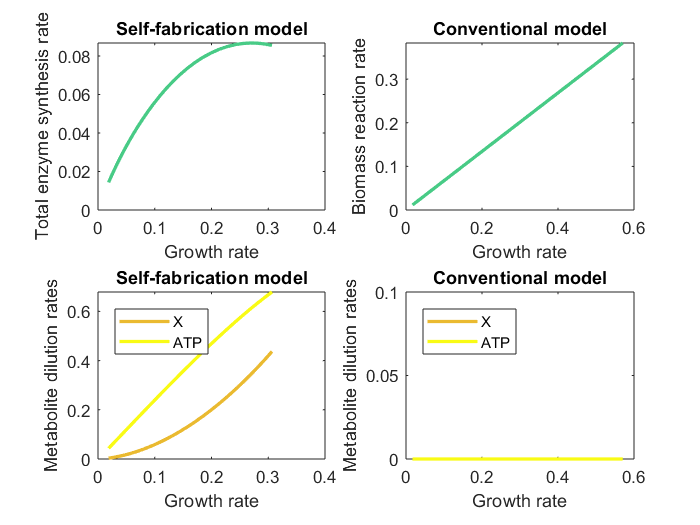

Supplement: S2 Code — (ZIP) [file pcbi.1007559.s011.zip › figures/biomass_proportionality_growth.png]
